# Influence of Acetylcholine Esterase Inhibitors and Memantine, Clinically Approved for Alzheimer’s Dementia Treatment, on Intestinal Properties of the Mouse

**DOI:** 10.3390/ijms22031015

**Published:** 2021-01-20

**Authors:** Vu Thu Thuy Nguyen, Jason Sallbach, Malena dos Santos Guilherme, Kristina Endres

**Affiliations:** Department of Psychiatry and Psychotherapy, University Medical Center of the Johannes Gutenberg-University Mainz, 55131 Mainz, Germany; VuThuThuy.Nguyen@unimedizin-mainz.de (V.T.T.N.); jsallbac@students.uni-mainz.de (J.S.); Malena.DosSantosGuilherme@unimedizin-mainz.de (M.d.S.G.)

**Keywords:** enteric nervous system, gut-brain-axis, microbiota, neurites, neurodegeneration

## Abstract

Four drugs are currently approved for the treatment of Alzheimer’s disease (AD) by the FDA. Three of these drugs—donepezil, rivastigmine, and galantamine—belong to the class of acetylcholine esterase inhibitors. Memantine, a NMDA receptor antagonist, represents the fourth and a combination of donepezil and memantine the fifth treatment option. Recently, the gut and its habitants, its microbiome, came into focus of AD research and added another important factor to therapeutic considerations. While the first data provide evidence that AD patients might carry an altered microbiome, the influence of administered drugs on gut properties and commensals have been largely ignored so far. However, the occurrence of digestive side effects with these drugs and the knowledge that cholinergic transmission is crucial for several gut functions enforces the question if, and how, this medication influences the gastrointestinal system and its microbial stocking. Here, we investigated aspects such as microbial viability, colonic propulsion, and properties of enteric neurons, affected by assumed intestinal concentration of the four drugs using the mouse as a model organism. All ex vivo administered drugs revealed no direct effect on fecal bacteria viability and only a high dosage of memantine resulted in reduced biofilm formation of *E. coli*. Memantine was additionally the only compound that elevated calcium influx in enteric neurons, while all acetylcholine esterase inhibitors significantly reduced esterase activity in colonic tissue specimen and prolonged propulsion time. Both, acetylcholine esterase inhibitors and memantine, had no effect on general viability and neurite outgrowth of enteric neurons. In sum, our findings indicate that all AD symptomatic drugs have the potential to affect distinct intestinal functions and with this—directly or indirectly—microbial commensals.

## 1. Introduction

Alzheimer’s disease (AD) is a devastating neurodegenerative disease that mainly affects the elderly population. Despite intense research effort, the underlying cause of the sporadic variant, which makes up 95% to 99% of all cases, has not been identified yet. This might be one explanation for the existing lack of curative and all the more preventive drugs—at least when used as monotherapeutics [1]. Until now, only medications for treatment of symptoms, such as memory loss evoked by hypo- or hyperactivity of single neurotransmitter pathways, are clinically available. These include acetylcholine esterase inhibitors (AChEI), such as donepezil and the NMDA receptor antagonist memantine.

The “cholinergic hypothesis” was one of the first evolving when attempting to clarify the origin of AD [2] and since then has been intensely studied. The concept is quite attractive as the nucleus basalis of Meynert, which represents the major source of cholinergic innervation of the cerebral cortex, is severely affected by neurodegenerative processes of the disease [3,4,5]. Moreover, cortical cholinergic changes display a topographic vulnerability that overlaps with important functional hubs (summarized in [6]) and usage of anti-cholinergic drugs evoked cognitive deficits (e.g., [7]; for a critical review on scopolamine-induced memory deficits as an AD model see [8]). Therefore, the development of AChEI offered a plausible therapeutic route due to their acetylcholine elevating effect. Donepezil received approval for AD treatment in 1996 for mild to moderate AD and in 2010 for the treatment of moderate to severe AD (for a recent overview on AChEI: [9]). A meta-analysis from 2017 reports its superiority against the two other approved drugs rivastigmine and galantamine (both approved in 2000 for the treatment of mild to moderate AD) concerning global AD symptomatology [10].

Besides the cholinergic transmission, NMDA receptor agonists were also suspected early on to act neurotoxically in the context of AD [11]. This led to development of memantine, a NMDA receptor antagonist, for therapeutic usage and approval by the FDA in 2003. The treatment results in a small clinical benefit (e.g., performance of activities of daily living) in patients with moderate to severe AD, while no significant difference in the placebo group was identified in mildly affected patients by a recent meta-analysis [12]. The effects of the AChEI are also described to be only modest and not long-lasting [13,14,15].

The most frequently encountered adverse effects of the cholinesterase inhibitor class as a whole are symptoms regarding the gastrointestinal tract [16]. Besides headaches, hypertension, and pain, constipation is one of the negative events reaching significant odds ratio caused by memantine treatment [17]. This is highly relevant, since within the last years, the importance of the gastrointestinal microbiome in neurodegenerative diseases evolved as a new field of research. Investigations comparing the gut microbiome of AD patients with age-matched healthy controls has not resulted in a conclusive finding (e.g., [18]). However, it is expected that a growing number of reports will respond to the question, whether our microbial commensals really impact CNS disorders, such as AD. While the impact of medication, including antibiotics and other drugs is intuitively assumed and experimentally demonstrated in microbiota studies (for example reviewed in [19]), also consistency in the stool has a significant effect on the microbiome [20]. Therefore, both should be carefully considered in line with such investigations. Nevertheless, a closer look at these parameters is missing for AD in regard to microbial analyses. For Parkinson’s disease, a disorder related to AD by its protein-deposits, taxonomic shifts were shown to be associated with stool consistency and constipation [21]. Moreover, the relative abundances of Faecalibacterium and Enterococcaceae were significantly influenced by L-DOPA medication [22]. This indicates that studies on AD would probably also benefit from taking medication and reported gastrointestinal disabilities into account.

To analyze a probable impact of AD approved drugs on the gastrointestinal system in general, data were extracted from http://drugcentral.org to collect relevant information regarding gastrointestinal tract (GIT) symptoms from the FDA Adverse Event Reporting System (Table 1). The likelihood ratio (LR) is the probability of a given adverse event (AE) in a patient with the target disorder divided by the probability of that same AE in a person without the target disorder. For all four drugs, the three AChEI and memantine, LRs above the respective threshold for GIT-relevant AEs were found: galantamine only elicited decreased appetite and vomiting as potential AE with increased LR. However, for donepezil, rivastigmine, and memantine multiple AEs within the categories eating behavior, dysfunction of the gut or the hepatic/pancreatic organ system could be identified. This indicates that patients with AD show certain vulnerability towards such GIT AEs. Additionally, this already implicates that the GIT must be a target tissue of the drugs and indeed, expression of cholinergic as well as of NMDA receptors within the gut has been reported throughout the whole length of the intestine.

Cholinergic neurotransmission has a key role in motility and secretory reflexes in addition to intrinsic sensory and vascular reflexes of the gut [23]. On enteric neurons, mainly α3β4 and α3β2 heteromers of nicotinic ACh receptors have been found, but also α7 homomers [24]. NMDA receptor chains NR1 and 2A and B have been identified on both, enteric neurons and glia, and muscarinic ACh receptors (mainly M1R to M4R) are widespread on different cell types in the GIT. Therefore, for example, deciphering the effect of AChEI on gut peristalsis is difficult, as nicotinic receptor activation is predominantly involved in enteric ascending reflex pathways, but cholinergic transmission is also involved in the descending inhibitory reflex due to muscarinic receptors [23]. Together with the reported AEs in patients, the expression of ACh, as well as NMDA receptors in the gastrointestinal system prompt a potential impact of the symptomatic drugs on physiological function of this organ system and in parallel or subsequently on its microbial inhabitants. Therefore, we here aimed at investigating if the four therapeutically applied drugs donepezil, rivastigmine, galantamine, and memantine display impact on crucial gut parameters, such as bacterial viability, biofilm formation, and colonic transit time, as well as properties of enteric neurons such as calcium-signaling or neurite outgrowth by using the mouse as a model organism.

## 2. Results

### 2.1. Effect of AChEI and Memantine on Viability of Fecal Bacteria and Biofilm Formation of E. coli

Several immune cells, such as macrophages, dendritic cells, and T cells are—in addition to neurons—affected by the release of neurotransmitters such as ACh (reviewed in [25]), which together with a probable altered peristalsis, makes a prediction towards microbiota within the complex environmental niche rather difficult. We decided to firstly investigate a potential direct effect of the respective medications on gut bacteria. Drug dosages to be administered were calculated by taking into account given information on plasma concentrations, intestinal excretion rates, and metabolic conversion (Table 2). Two concentrations per drug were finally chosen (high and low) for investigating the impact on bacterial viability. Not all applied concentrations were above the IC_50_; however, all of them were in the order of magnitude of this parameter.

**Figure 1:**
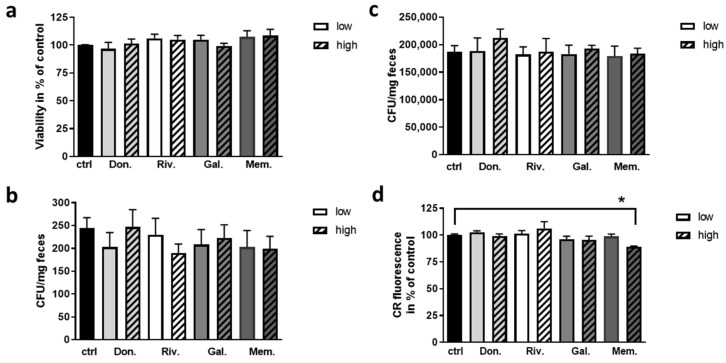
Effect of AD therapeutic drugs on growth and biofilm formation of bacteria. (**a**) Fresh fecal samples from wild type C57BL6/J mice were collected, diluted appropriately and incubated with the respective drug for 10 min (ctrl: DMSO; Don.: donepezil; Riv.: rivastigmine; Gal.: galantamine; Mem.: memantine; for the concentrations see Table 2). (**a**) ATP content was measured with the BacTiter-Glo assay (*n*= 4; for each measurement performed in technical duplicates, fecal samples from 4 donor animals each were pooled; 50% females). Suspensions of fecal material were additionally plated after the incubation with the respective drug on family-specific plates. Colony forming units (CFU) were counted after 20 h of incubation for Enterobacteriaceae (**b**) and Lactobacillaceae (**c**). (**d**) Biofilm formation of *E. coli* was measured by assessment of the fluorescent signal of Congo red (CR) binding to amyloidogenic fibers within the biofilm derived by cultivation on YESCA-agar. Data are presented as mean + SEM. Statistical analysis was conducted by One Way ANOVA with Sidak’s multiple comparison test (* *p* < 0.05).

Firstly, impact of acute treatment with the two drug dosages for fecal bacteria was assessed (Figure 1a). None of the drugs revealed significant change of bacterial viability, as measured by ATP content, nor occurred a difference between the applied dosages. As the acute treatment of 10 min might not be sufficient to evoke toxic effects or these might only influence single bacterial families, subsequent colony forming was investigated for Enterobacteriaceae and Lactobacillaceae from murine feces (Figure 1b,c). Here, no adverse effects could be observed.

The number and viability of microbiota might be of importance for the interaction with the host. Additionally, the production of certain metabolites or growth condition might be relevant such as biofilm formation [29]. Therefore, *E. coli* as a bacterium, which is also present in murine gut (for example, [30]) was plated after incubation with the drugs on biofilm-promoting YESCA-agar and biofilm formation was assessed by Congo red stain (Figure 1d). None of the applied AChEIs elicited impairment of biofilm formation, while the high concentration of memantine resulted in a reduction of about 10% (*p* = 0.026).

### 2.2. Impact of AChEI and Memantine on Intestinal Properties of the Mouse

**Figure 2:**
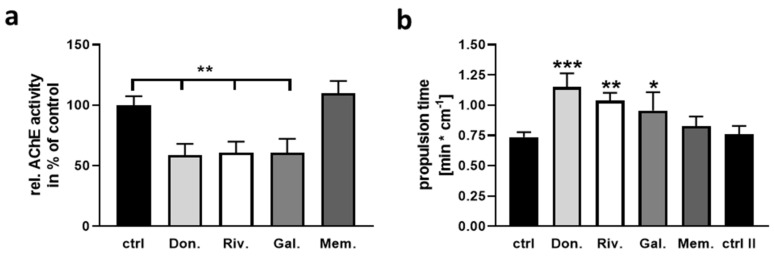
Assessment of colonic AChE activity and propulsion time upon application of AChEI or memantine. Pieces of proximal colon from wild type mice were dissected, cleaned, and exposed for 1 min to DMSO with a subsequent second exposure to either drug or a second DMSO application. (**a**) Enzymatic activity was measured using Ellmans reaction after propulsion time measurement in the ex vivo organ bath (*n* = 6 per group with three donor mice). Propulsion time was recorded by inserting a dried fecal pellet from the respective mouse and camera-based time-lapse measurement ((**b**), *n* = 5 per group or *n* = 4 (DMSO-double incubation, ctrl II)). Data are presented as mean + SEM. Statistical analysis was conducted by One Way ANOVA with Sidak’s multiple comparison test (* *p* < 0.05, ** *p* < 0.01, *** *p* < 0.001).

The cholinergic system has a dominant role in gut propulsion and other properties of the gut (for a review on vertebrate gut motility: [31]). To assess the theory that concentrations achieved within the gut lumen can act on the activity of gut acetylcholine esterase, we measured the enzyme’s activity within colon samples with a 1 min incubation using the high dosages (see Table 2). All three AChEIs resulted in a significantly reduced enzyme activity while memantine had no effect (Figure 2a). To investigate the impact of this inhibition in a proxy for the physiological context, an ex vivo organ bath was used (modified from [32]). Pieces of proximal colon were exposed to DMSO as solvent control, followed by a 1 min incubation with the respective drug. To ascertain that this subsequent inter-individual measurement does not affect the tissue’s reaction in the second measurement session due to longer time ex vivo, a two-time exposure with DMSO was conducted in some specimens (ctrl II in Figure 2b). This control revealed that no general change in propulsion was obtained between a first and a second measurement step. All three AChEI led to a significantly elongated propulsion time (for example 1.5 fold for donepezil) while memantine remained without effect.

### 2.3. Effect of AChEI and Memantine on Calcium Signaling and Neurite Outgrowth of Enteric Neurons

**Figure 3:**
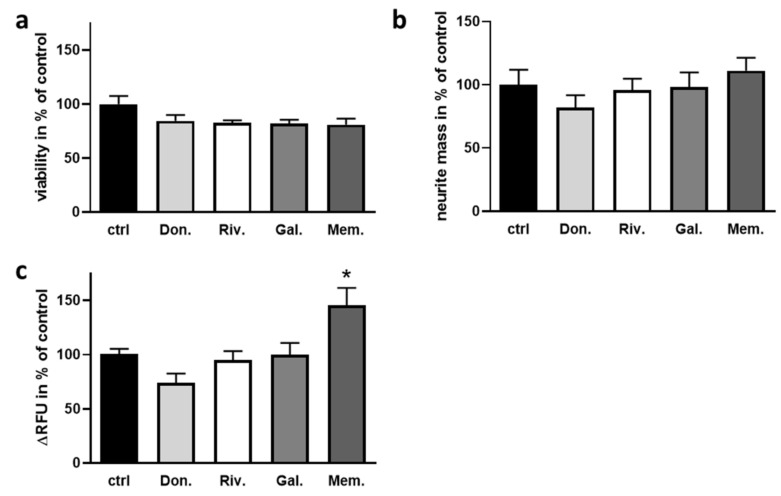
Influence of AChEI and memantine on enteric neurons. (**a**) Neurons were treated on DIV3 for 24 h with the indicated drug or DMSO as a solvent control (ctrl). Viability was analyzed by the CellTiter Glo assay (*n* = 4 donor C57B6L/J wild type mice, aged 2–3 months). (**b**) Neurite mass was assessed after 24 h growth with the respective drug. Cells were cultivated on transwell plates and neurite mass determined after stripping off cell bodies by crystal violet stain (*n* = 4 donor mice). (**c**) Calcium influx into enteric neurons treated for 25 min with the respective drugs or DMSO was measured by calcium-stain after KCl injection into the medium. Slope of the fluorescent signal in % of the solvent control is shown. (*n* = 8–11 per group, 4 donor mice). Data are presented as mean + SEM. Statistical analysis was conducted by One Way ANOVA with Dunnett’s multiple comparison test (* *p* < 0.05).

To further analyze the impact of the drugs approved for AD on characteristics of enteric neurons, primary cultures were prepared and calcium signaling in the presence of the respective drugs (high dosage, see Table 2) measured. An ATP-dependent viability assay firstly confirmed that the concentration of the applied drugs did not evoke neurotoxic effects by 24 h of incubation (Figure 3a). Neurite outgrowth was measured by crystal violet stain of neurites grown through a transwell mesh and revealed no impact of the AChEI or memantine on neurite mass due to 24 h of incubation with the respective drugs (Figure 3b). Subsequently, calcium influx after hypopolarization due to potassium chloride treatment was quantified with a fluorescent calcium-binding stain in enteric neurons submitted to 25 min of treatment with the drugs (Figure 3c): none of the AChEI resulted in a significantly altered calcium-dependent signal while memantine elicited an increase of about 50%.

## 3. Discussion

Gastrointestinal symptoms have been described for all AChEI and for memantine in treatment of AD (for example [33]), but additionally, in other therapeutic approaches (e.g., donepezil in autism in younger patients, [34]). While the importance of the cholinergic system for gastrointestinal properties is well-accepted (e.g., [35]), it has not been analyzed so far, including what exactly causes the complications of treatment in detail. A direct effect on neurons of the enteric nervous system might be assumed, as well as the effect on immune cells or bacterial commensals. Moreover, aggravating effects of already pathology-derived gastrointestinal symptoms are also possible. For untreated AD, no structured report on gastrological disease symptoms is available; however, problems with swallowing have been repeatedly found [36]. In addition, dysbiosis seems to occur—even if the outcome of the studies so far is rather inconsistent [18]. For Parkinson’s disease (PD), which is related to AD for many reasons, both, gastrointestinal dysfunction, as well as changes in microbiota have been already described for years (reviewed for example in [37,38]). More recently, an influence of medication with L-DOPA and entacapone on the relative abundance of e.g., the bacterial genera Bifidobacterium and Ruminococcus were demonstrated for PD [22]. This together with the fact that bacterial tyrosine decarboxylases restrict bioavailability of L-DOPA in plasma of experimental animals [39] underlines the intimate relation of medication and bacterial commensals in PD. For AD, scarce data are available.

Here, we investigated the impact of approved AD drugs on bacterial commensals and enteric neurons in a concentration range that might be approximately available within the intestinal lumen. Tissue specimen and fecal bacteria from a commonly used mouse strain (C57BL/6J) were used in this regard. Mouse and human gut physiology as well as anatomical structures are quite similar even if some differences are observed, such as presence of an appendix or villus length in the small intestine (for a detailed comparison of human and murine gut see [40]). Comparability of the microbiome of both species is not unmitigated. However, the usage of murine models for diseases, such as inflammatory bowel disease has provided useful knowledge despite all limitations (e.g., [41,42]).

Viability of murine fecal bacteria was not mainly affected by a 10 min exposure to the AD approved drugs or after plating. Biofilm formation was also not influenced by the AChEI, while memantine decreased biofilm mass by about 10%. We are not aware of any report about interference of memantine or any of the AChEI with bacterial biofilm formation within another research context. Nevertheless, memantine has been reported to exhibit anti-aggregation properties on A-beta [43]. Comparably to A-beta, some compounds of bacterial biofilms present amyloidogenic properties such as curli [44]. Therefore, interaction of the drug with this bacterial protein secreted during biofilm formation is in principle conceivable. However, mM dosages of memantine were needed to evoke the described effects on A-beta, while here the highest dosage applied was 0.2 µM.

Anti-microbial functions have only been occasionally reported for the AChEI or memantine as e.g., an inhibition of viral replication by donepezil in leukocytes [45]. Memantine for example blocks bacteremia and meningitis in mouse models [46]. This was due to impaired intracellular survival of bacteria but not related to inhibition of extracellular bacterial viability. It was speculated that modulation of both, inflammatory and anti-inflammatory pathways of the host, was underlying these observations. Likewise, memantine was found to promote trapping and bactericidal activity of neutrophils of the host [47].

Glia cells that have not been addressed in the here presented investigation might also be drivers of the observed influence of the AChEI on colonic motility since they are able to detect ACh e.g., via M3 and M5 subtypes of muscarinic receptors [48]. With the administered drug dosage, around 50% reduction of AChE activity were reached in colon tissue preparations and about 50% of propulsion time increase, for example for donepezil. Such a reduced transportation rate of the gut content alone can lead to changes in experimentally measured microbiota in patients under therapy. Using the Bristol stool scale, Vandeputte and colleagues came to the final conclusion that human stool consistency strongly correlated with all investigated microbiome markers [20]. Stool consistency, in turn, is strongly depending on transit time due to duration of water resorption. This should be mandatorily taken into account when comparing microbiomes derived from differently medicated AD patients.

In relation to the enteric neurons, the AChEI in the respective concentration did neither affect viability of the neurons nor neurite mass or calcium signaling. For donepezil, a neurite outgrowth promoting property has been described before, e.g., for PC12 cells [49]. However, the effect was found synergistically to NGF treatment and in a dosage of 10 µM which exceeds the here administered drug dosage by factor 1000. Similarly, memantine was found to increase neurite length in cortical cultures to about 130% of control at 1 µM [50]. Here, we used maximally 0.26 µM. While calcium influx was not affected by any of the AChEI, memantine significantly increased it by 40%. Memantine—as a NMDA receptor antagonist—should normally attenuate this when the signal is glutamate-evoked (e.g., [51]). However, here we used activation of neurons by KCl and memantine was described to increase the content of the intracellular calcium store by potentiating the activity of the SERCA pump, and thereby, e.g., carbachol-activated calcium influx [52].

Our investigation in sum provides evidence that the direct impact of AChEI on the gut microbial commensals and enteric neurons is rather restricted in the estimated dosages present in the gut, while memantine had slight but significant effects. Whether locally higher concentrations might be found that exert a direct influence cannot be deduced from the here presented experiments.

Further limitations of our study are given by the fact that all investigations were done in wild type mice without pathological symptoms. As the AD-typical A-beta peptide directly interferes with the host’s microbiota [53,54], we cannot exclude that on an already dysfunctional background, a different outcome will be observed. The drugs used in this investigation show effectiveness on AD-like symptoms in rodent disease models such as the 5xFAD strain [55,56]. However, at least bacterial viability, as well as cultivation of Lactobacillaceae and Enterobacteriaceae remained similarly unaffected for feces derived from 5xFAD mice under drug administration as observed for samples from wild type mice (Figure S1). Together with long-term applications, investigations regarding the influence of symptomatic anti-AD drugs and especially the AChEI on the intestinal cholinergic anti-inflammatory pathway [25,57] need to be conducted in the future.

## 4. Materials and Methods

### 4.1. Animals

C57BL/6J OlaHsd mice (Envigo, Gannat, France) were single-housed one week before start of the experiment to prevent coprophagy and stress due to destroying the group composition by sacrifice of single animals. Mice were maintained with a 12-h day/night cycle. Food and water were available ad libitum. The mice used for the respective experiments were between 34 and 41 weeks of age despite the mice for enteric neuron primary culture (aged 8–12 weeks).

### 4.2. Determination of Fecal Bacteria Viability

Freshly excreted fecal pellets of four mice per experiment (two females and two males) were collected and suspended in isotonic sodium chloride solution (0.9%, 100 μL per mg feces). The suspensions obtained from each mouse were mixed in equal parts and stored on ice. To 1 mL of suspension 1 μL of the respective drug dilution was admitted and mixed. A solvent control was set up in the same manner by using DMSO. The obtained suspensions were incubated for 10 min at 37 °C and inverted every 2 min.

### 4.3. Cultivation of Enterobacteriaceae and Lactobacillaceae and Determination of CFU

To assess the influence of the investigated drugs on the microbial families of Enterobacteriaceae and Lactobacillaceae, dilutions of bacterial suspensions (see above) with isotonic sodium chloride were prepared as follows: for plating of Enterobacteriaceae 0.27 mg feces/mL, for plating of Lactobacillaceae 0.001 mg feces/mL. 1 mL of these suspensions was plated on 3M^TM^-Petrifilm plates for either Enterobacteriaceae or Lactobacillaceae (3M Deutschland GmbH, Neuss, Germany). The plates were incubated for 20 h at 37 °C. Formed colonies were counted and the number of colony forming units (CFU) per mg feces calculated.

### 4.4. Bacterial Viability Assay

A 1:100 dilution of the prior prepared fecal suspensions (see above) was produced for each sample. Viability was measured by using the BacTiter-Glo^TM^-assay as described by the vendor (Promega GmbH, Mannheim, Germany). As a background control, isotonic sodium chloride solution was used. Luminescence was measured in white 384 well plates by using the FluoStar Omega (BMG Labtech, Cary, NC, USA).

### 4.5. Production of Bacterial Biofilms

For the production of biofilms, cultures of *E. coli* (DH5alpha, NEB Biolabs, Ipswich, Massachusetts, USA) were used. 10 μL of *E. coli*-culture were diluted in 5 mL LB medium and incubated for 12 h at 37 °C and 300 rpm. OD_600_ was measured and the suspension diluted with LB medium to an OD_600_ of 0.1. Incubation was conducted for 2 h at 37 °C and 300 rpm under anaerobic conditions by purging with argon gas. After the incubation period, 5 μL of a dilution of the respective drug (Table 1) in LB medium was added to 50 μL suspension each. Samples were purged with argon and kept at 37 °C for 2 h at 300 rpm. The suspensions were diluted 1:10 and 20 μL per well added to a black 96-well plate previously coated with 140 μL YESCA-agar. The plate was purged with argon, sealed, and kept at 28 °C for 48 h.

### 4.6. Staining of Biofilms Using Congo Red

To each well containing biofilms 60 μL Congo red solution (Sigma Aldrich, St. Louis, MO, USA, 1 mg/mL H_2_O) were added and incubated at room temperature for 15 min. The supernatant was carefully removed and 70 μL sodium chloride (1 M) added per well. The supernatant was aspirated after 15 min and the washing step was repeated. The fluorescence signal was measured at an excitation wavelength of 485 nm and an emission wavelength of 620 nm.

### 4.7. Determination of Colon Transition Time Ex vivo

Mice were anesthetized using isoflurane and sacrificed by decapitation. The colon was dissected and stored in Krebs-buffer at 37 °C. A modified version of the experimental setup named “Gastrointestinal Motility Monitor” [58] was used to determine the colon transition time. Therefore, the colon was first incubated in Krebs-buffer containing DMSO (1 μL DMSO/ mL Krebs-buffer) at 37 °C for 1 min. The tissue was rinsed with Krebs-buffer and fixed in an organ bath filled with continuously carbogen-gas fumigated Krebs-buffer at 37 °C. A continuous flow of Krebs-buffer was provided by a pump (DC 308, Anself, Portland, OR, USA) where the direction of flow coincided with the alignment of the colon from oral to anal. A fecal pellet of the respective mouse was inserted in the oral end of the colon and incubated for 3 min. Subsequently, the pellet was carefully pushed a small distance towards the anal end until active propulsion could be observed. The process of movement was recorded with a video camera (COOAU, Seattle, Washington, DC, USA). Afterwards, the same colon specimen was rinsed with Krebs-buffer, incubated in a dilution of the respective drug in Krebs-buffer (1 μL/ mL) for 1 min at 37 °C and transition time measured again as described. To ascertain that the colon showed a comparable activity between the first and the second measurement, in some cases DMSO was also used a second time, designated then as ctrl II.

### 4.8. Acetylcholinesterase Activity Measurement

Acetylcholine esterase activity in colon samples (see 4.7.) was determined following the method of Ellman as described before [59]. In brief, colon tissue was homogenized with potassium phosphate buffer (0.5 M) containing Protease inhibitor (Roche, Basel, Switzerland) followed by sonication. Homogenates were subsequently centrifuged at 4 °C for 1 min at 1000 g. Supernatants were diluted 1:10 with buffer and acetyl thiocolineiodid (Sigma-Aldrich, St. Louis, MO, USA) (1 mM) and 5,5′-Dithiobis-2-nitrobenzoic acid (Sigma-Aldrich) (0.25 mM) added. Absorption at 405 nm was measured after 5 min incubation in the dark, slope for the enzymatic reaction calculated and values normalized to DMSO control.

### 4.9. Isolation and Cultivation of Enteric Neurons

The isolation and cultivation of enteric neurons were carried out as described before [59]. Cells were cultivated in a 96-well plate (Greiner Bio-One International GmbH, Kremsmünster, Austria) coated with Poly-L-Lysine. 15 μL neuron-containing suspension from tissue preparations were added to 112.5 μL of neuronal growth medium. Plates were kept at 5% CO_2_, 37 °C and 95% humidity. On the third day of cultivation (DIV3), half of the medium was replaced by 36.25 µL of fresh medium supplemented with drug or DMSO for the viability assay or only fresh medium for the calcium assay. After 24 h (DIV4), calcium measurement after 15 min drug exposure or viability assay were carried out.

### 4.10. Viability Assay for Primary Enteric Neurons

Potential cytotoxic effects of the drugs were assessed by using the CellTiter-Glo Assay (Promega, Mannheim, Germany) in 96-well formats (white, flat glass bottom, Greiner Bio-One International GmbH, Kremsmünster, Austria). The assay was performed according to the method description in [60].

### 4.11. Determination of Neurite Mass

Neurite outgrowth was analyzed by a transwell-system in combination with crystal violet stain as described previously [60]. In brief, cell culture transwell inserts (3 micron pore size, 24-well formats, Nunc™, Thermo Scientific, Karlsruhe, Germany) were coated with extracellular matrix gel from Engelbreth-Holm-Swarm murine sarcoma (Sigma Aldrich). Neurons were seeded on the inserts and treated on DIV3 for 24 h with the respective drug or DMSO (solvent control). Afterwards, neuronal cell bodies from the upper surface of the insert were removed with a cotton swab and neurites stained with crystal violet solution (Sigma Aldrich). The stain was extracted, quantified at 595 nm, and normalized to OD340 (Biochrom ASYS Hitech Expert 96 UV Microplate Reader).

### 4.12. Calcium Influx Measurement

Calbryte 520-AM (AAT Bioquest 20651, Sunnyvale, CA, USA) was used to measure the calcium influx upon KCl administration. 62.5 μL supernatant was removed from each cell-containing well. To each well, 50 μL of 10 μM Calbryte^TM^-solution in DMSO were added and plates kept at 37 °C in the dark for 45 min. After 35 min, 1 μL of the respective drug (Table 2) in HEPES-buffer was added. DMSO was used as the solvent control. After incubation for 45 min, the plate was kept another 15 min at room temperature in the dark and was then washed with 100 μL HEPES-buffer per well. The slope of fluorescence signal increase evoked by calcium influx upon addition of 50 mM potassium chloride was calculated as described previously [59].

### 4.13. Statistical Analysis

GraphPad Prism 8 for Windows was used for statistical analyses. The results are graphically presented as mean + standard error of the mean (SEM). The results of the investigated drugs were compared to the DMSO solvent control by using One-way analysis of variance (ANOVA) where *p*-values < 0.05 were considered statistically significant (*: *p* < 0.05; **: *p* < 0.01; ***: *p* < 0.001).

## Figures and Tables

**Table 1:** Gastrointestinal adverse events. Relevant symptoms for the four investigated drugs were selected from http://drugcentral.org/ (LR: Likelihood ratio).

Category	MedDRA Adverse Event Term	LR Donepezil	LR Rivastigmine	LR Galantamine	LR Memantine
Eating behavior	Decreased appetite	335.24	127.75	67.45	199.77
	Dehydration	154.16	134.20	-	160.69
	Diet refusal	-	-	-	44.01
	Dysphagia	-	145.45	-	63.51
	Eating disorder	-	44.27	-	48.36
	Hypophagia	63.10	-	-	43.76
	Malaise	-	280.04	-	105.56
	Nausea	144.38	315.48	-	77.11
	Salivary hypersecretion	53.98	-	-	-
	Weight decreased	54.62	124.86	-	89.91
Gastrointestinal dysfunction	Anal incontinence	48.70	-	-	-
	Constipation	-	-	-	76.09
	Diarrhoea	163.56	132.59	-	-
	Faecaloma	-	-	-	40.77
	Gastric ulcer	62.65	-	-	
	Gastrointestinal haemorrhage	99.75	41.61	-	-
	Gastrointestinal disorder	-	65.65	-	-
	Duodenal ulcer	79.33	-	-	
	Melaena	43.41	-	-	-
	Upper gastrointestinal haemorrhage	49.68	-	-	-
	Vomiting	332.71	438.03	55.85	104.10
Pancreatic/hepatic dysfunction	Aspartate aminotransferase ↑	-	-	-	68.31
	Alanine aminotransferase ↑	-	-	-	56.78
	Blood alkaline phosphatase ↑	-	-	-	68.56
	Hepatic function abnormal	63.00	-	-	-
	Hepatitis	-	47.10	-	56.62
	Pancreatitis	-	-	-	53.69
	Pancreatitis acute	-	-	-	97.25

↑: elevated.

**Table 2:** Estimation of fecal drug concentration. Putative fecal concentrations of the four drugs were calculated by using reported fecal excretion rates. In case only urinary excretion has been investigated, remaining percentage was assumed to be excreted by the gastrointestinal tract. Available ratios of unchanged drug excretion were also taken into account to calculate minimally amounts (low). (nr: not reported; information taken from [26,27,28] and Novartis drug information; https://www.drugs.com/ppa). (BChE: Butyrylcholine esterase).

Reported Parameters	Donepezil	Rivastigmine	Galantamine	Memantine
Plasma concentration [ng/mL]	29.38	8.7 (oral) 21.6 (patch)	83.7	179.3
IC_50_ [ng/mL]	2.55 (AChE)	1.04 (AChE) 9.25 (BChE)	1.62 (AChE/BChE)	97 (NMDAR)
Urinary excretion	57%; 17% as unchanged drug	97% as metabolites	60–93%	74%; 48% as unchanged drug
Fecal/ intestinal excretion	15%	less than 1%	nr	nr
Deduced dosages for the following experiments [ng/mL]	1.3 (low) 4.5 (high)	0.2 (low) 0.9 (high)	5.9 (low) 33.5 (high)	22.4 (low) 46.6 (high)

## Data Availability

The data presented in this study are available in the article.

## Supplementary Materials

The following are available online at https://www.mdpi.com/1422-0067/22/3/1015/s1, Figure S1: Effect of AD therapeutic drugs on growth of fecal bacteria from 5xFAD transgenic mice.
