# Supplementary material for: Influence of Acetylcholine Esterase Inhibitors and Memantine, Clinically Approved for Alzheimer’s Dementia Treatment, on Intestinal Properties of the Mouse
Source: Int J Mol Sci. 2021 Jan 20;22(3):1015. doi: 10.3390/ijms22031015 (PMC7864027; doi:10.3390/ijms22031015)
Supplement: Supplementary file 1 [file ijms-22-01015-s001.pdf]

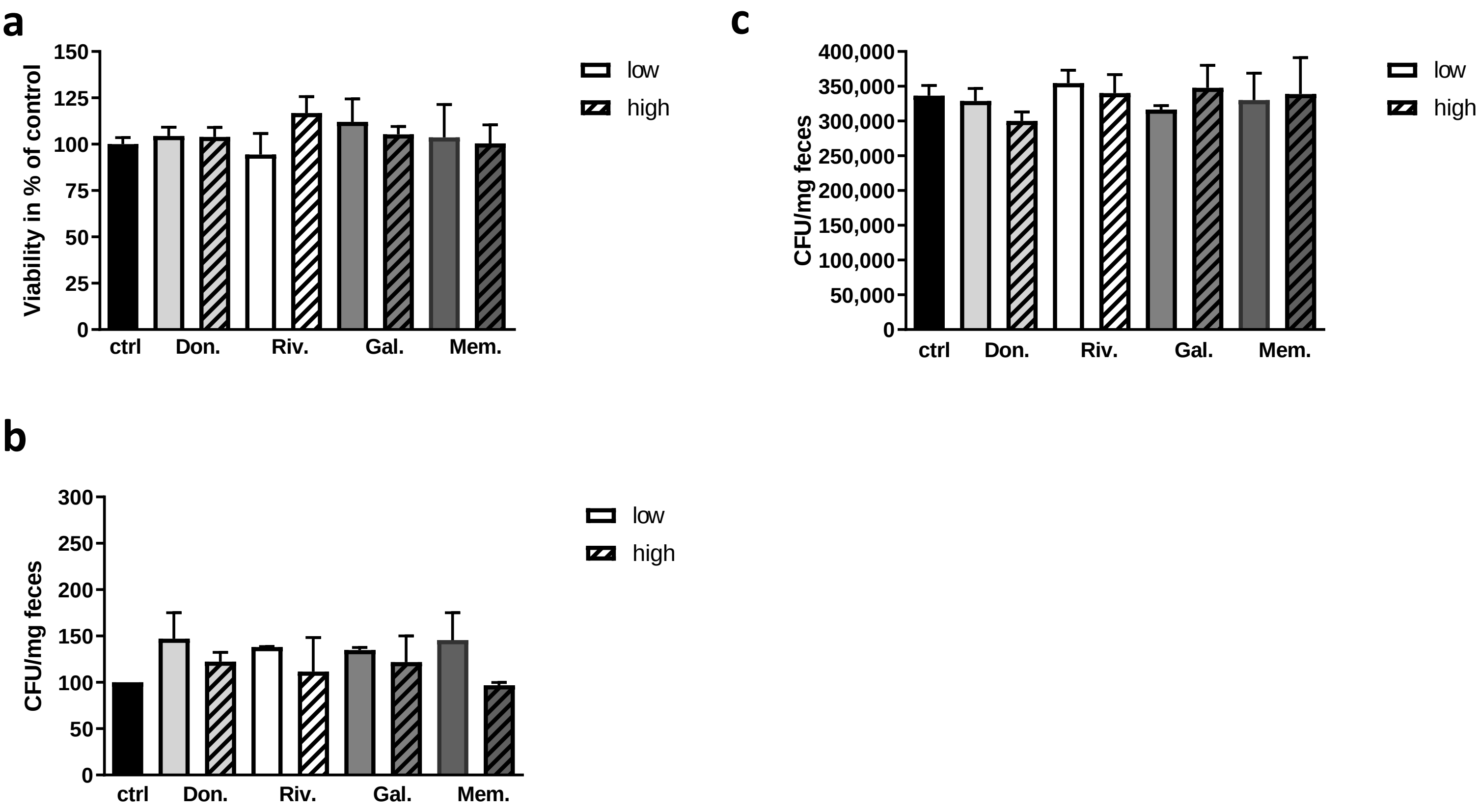

Suppl. Figure 1. Effect of AD therapeutic drugs on growth of fecal bacteria from 5xFAD transgenic mice. (a) Fresh fecal samples from 5xFAD mice (aged 34-40 weeks) were collected, diluted appropriately and incubated with the respective drug for 10 min (ctrl: DMSO; Don.: donepezil; Riv.: rivastigmine; Gal.: galantamine; Mem.: memantine; for the concentrations see Table2). (a) ATP content was measured with the BacTiter Glo assay (n= 2; for each measurement performed in technical duplicates, fecal samples from 4 donor animals each were pooled; 50% females). Suspensions of fecal material were additionally plated after the incubation with the respective drug on family-specific plates. Colony forming units (CFU) were counted after 20 h of incubation for Enterobacteriaceae (b) and Lactobacillaceae (c). (Data are presented as mean + SEM. Statistical analysis was conducted by One Way ANOVA with Sidak's multiple comparison test.
